# Developmental trajectories of tobacco use and risk factors from adolescence to emerging young adulthood: a population-based panel study

**DOI:** 10.1186/s12889-022-14070-3

**Published:** 2022-08-29

**Authors:** Seong Yeon Kim, Sung-il Cho

**Affiliations:** 1 Department of Public Health Science, Graduate School of Public Health, Seoul National University, 1 Gwanak-ro, Gwanak-gu, Seoul, 08826 Republic of Korea; 2 Institute of Health and Environment, Seoul National University, Seoul, 08826 Korea

**Keywords:** Adolescent, Smoking trajectories, Mobile phone dependency

## Abstract

**Background:**

Adolescence to young adulthood is a critical developmental period that determines lifelong patterns of tobacco use. We examined the longitudinal trajectories of tobacco use, and risk factors for its use, and explored the association between the trajectories of mobile phone dependency and smoking throughout the life-course among adolescents and young adults.

**Methods:**

Data of 1,723 subjects (853 boys and 870 girls) were obtained from six waves of the Korean Children and Youth Panel Survey (mean age = 13.9–19.9 years). To identify trajectories of smoking and mobile phone dependency, group-based trajectory modelling (GBTM) was conducted. A multinomial logistic regression analysis was performed to identify the characteristics of the trajectory groups.

**Results:**

GBTM identified four distinct smoking trajectories: never smokers (69.1%), persistent light smokers (8.7%), early established smokers (12.0%), and late escalators (10.3%). Successful school adjustment decreased the risk of being an early established smoker (odds ratio [OR] 0.46, 95% confidence interval [CI] 0.27–0.78). The number of days not supervised by a guardian after school was positively associated with the risk of being an early established smoker (OR 1.96, 95% CI 1.23–3.13). Dependency on mobile phones throughout the life-course was positively associated with the risk of being a persistent light smoker (OR 4.04, 95% CI 1.32–12.34) or early established smoker (OR 8.18, 95% CI 4.04–16.56).

**Conclusions:**

Based on the group-based modeling approach, we identified four distinctive smoking trajectories and highlight the long-term effects of mobile phone dependency, from early adolescence to young adulthood, on smoking patterns.

**Supplementary Information:**

The online version contains supplementary material available at 10.1186/s12889-022-14070-3.

## Background

Tobacco use is the leading cause of preventable death and a major public health challenge worldwide [1]. In an attempt to end the tobacco epidemic, many countries have focused on young people [1]. Adolescence to young adulthood is a critical developmental period that determines lifelong patterns of tobacco use [2, 3]. Most adult smokers start smoking during adolescence and young adulthood when people are vulnerable to social and environmental pressures [1, 4]. Especially, emerging adulthood is a period of life transition; young adults experience many changes, including in residence, peer groups, responsibilities, and so on [5]. Therefore, it is critical to understand smoking patterns during this period [2–5].

Longitudinal analyses of behavior focus on the transitions to figure out when they diverges [6]. Traditionally, hierarchical modeling and latent curve analysis are applied to identify patterns of health-related behaviors in longitudinal data. More recently, however, group-based trajectory modeling (GBTM) analysis has been used [7–10]. The former analyses estimate population average trajectories based on continuous distributions, whereas GBTM can accommodate a number of different distributions, including Poisson, zero-inflated poisson (ZIP), censored normal, and binary distributions [9]. GBTM clusters individuals with similar longitudinal trajectories according to an outcome of interest [6, 9]. It assumes that the population is composed of distinct groups showing similar progression over age or time, and imposes the same error variance for all classes and time points [11]. Group-based approaches are more suited for analyzing multinomial trajectories [9].

Many trajectory analyses have explored substance use over time. However, few studies have identified smoking trajectories from adolescence to emerging adulthood, or associated risk factors [2, 4, 5, 12]. White et al. (2002) explored smoking trajectories from the age of 12 to 30 years using GBTM, and identified three trajectory groups: heavy/regular, occasional/maturing out, and non/experimental smokers [2]. Similarly, Riggs et al. (2007) reported four smoking trajectory groups, from the age of 12 to 28 years, using GBTM: abstainers, low users, late stable users, and early stable users [12]. Dutra et al. (2017) explored smoking trajectories from the age of 12 to 30 years using latent class growth analysis, and identified five trajectory groups: never smokers, quitters, early established smokers, and late escalators [4].

Despite the high rate of mobile phone use in adolescents, little is known of the effect thereof on smoking patterns [13]. In South Korea, adolescents who own mobile phone have continued to increase, reaching over 90% as of 2018. Especially, the rate of smartphone ownership have begun to rise significantly from 2012 and reached 80% in 2018 [14]. The recent survey in 2020 reported that about 30.2% of adolescents and 33.7% of adults are showing overdependence on smartphone [15]. Also, Kim et al. (2018) reported that the dependency increases rapidly from the senior grades of elementary school. Although mobile phones including smartphones are useful in our daily lives, but overuse can also have adverse effects such as psychological problems as well as other developmental problems, especially for young people who are vulnerable to social environments [16, 17]. Several previous researches based on the cross-sectional study have shown that the problematic smartphone use is associated with earlier and more extensive adolescent substance use [18–20]. The studies reported that mobile phone use and online social networking increased the risk of substance use including smoking among adolescents [18, 21]. In addition, mobile phone dependency increases exposure to online images of substance use by peers, and to advertisements of various tobacco products [18, 22]. However, little is known regarding the longitudinal effects of mobile phone dependency on smoking patterns throughout the life-course among adolescents and young adults.

Developmental trajectory studies are based on the life-course approach; *i.e.*, study of the long-term effects of physical and social exposures from childhood to later adulthood [23, 24]. However, most longitudinal trajectory studies are limited to examine baseline predictors of trajectories. Therefore, we used a life-course approach to examine not only mobile phone dependency at baseline, but also the trajectories of mobile phone dependency from adolescence to young adulthood. We focused on the associations between the trajectories of mobile phone dependency and tobacco use.

We conducted a GBTM analysis with three objectives: to identify developmental smoking trajectories from early adolescence to emerging young adulthood; to examine the factors associated with smoking trajectories; and to explore the association between the trajectories of mobile phone dependency and smoking throughout the life-course among adolescents and young adults.

## Methods

### Data source and study population

Data were drawn from a Korean national cohort study, the Korean Children & Youth Panel Survey (KCYPS), conducted by the National Youth Policy Institute. This longitudinal survey has been administered annually from 2010 to 2016 to monitor individual development including health-related behaviors and environment of children and youths over time. By leveraging stratified multi-stage cluster sampling methods, the KCYPS selects nationally representative sample of Korean adolescents and is composed of three cohorts: the first and fourth grades of elementary school and the first grade of middle school. The survey was conducted at randomly selected schools by stratifying into 16 administrative districts. One class was randomly selected and all students in the selected class conducted interviews with interviewers. However, if more than 80% of the student questionnaire was non-responded due to the student’s disability or disease, it was excluded from the final data. Questions that were difficult for students to answer directly, such as household income, were measured through a telephone interview with the parents.

To demonstrate developmental smoking patterns from adolescence to young adulthood, this study selected the cohort data for first grade middle school students (*n* = 2,351; 78 schools). The study population began the survey at 12–15 years old (the 1^st^ grade), then they were followed up until 18–21 years of age. The response rate for the final survey wave in 2016 was 80.0% (*n* = 1,881). For the study, the last follow up point represents a young adulthood period, when most participants attended college or began their career. For the GBTM analysis, only respondents who participated in the final wave were included. Because the first wave in 2010 did not include a survey on smoking status, those data were excluded from the trajectory analysis. To reduce bias, along with the outcome variable, the data from the second wave in 2011 were used as the baseline measure for independent variables including age, gender, family income, number of days not supervised by a guardian after school, smoking friends, drinking experience, school adjustment, experience of health-related education, and mobile phone dependency. Subjects with missing sample weight values, and those who had not provided information on smoking status during all waves, were excluded. To account for the missing covariates, multiple imputation was employed. Overall, the study population comprised 1,723 subjects (853 males and 870 females).

### Measurements

#### Tobacco use

Smoking experience and frequency were measured during the survey. If the subjects smoked occasionally within a year, they reported the smoking frequency in the past year. If the subjects smoked regularly, they reported the daily smoking frequency. Since most subjects were nonsmokers and the distribution of smoking frequency was skewed, we categorized subjects as ‘nonsmokers’ (no smoking within the past year), ‘experimenters’ (smoked occasionally within the past year), ‘daily smokers’ (smoked 1–9 times per day), or ‘heavy daily smokers’ (smoked > 10 times per day) [25]. Through the GBTM, this study used the smoking trajectory groups as the outcome variables.

#### Covariates

Sociodemographic, environmental, and intrapersonal characteristics were examined. Age, gender, family income at baseline (wave 2), type of high school, and college status at the last follow-up (aged 18–21 years) were the sociodemographic factors. Age at baseline was adjusted for the analyses. Family income was classified as low (tertile 1), medium (tertile 2), or high (tertile 3). Each categories of family income approximately ranged from less than 35 Million Won, 35–49.99 Million Won, to 50 Million Won and above. Type of high school, as measured in wave 4, was included as a dichotomous variable (general/vocational). The general high schools include all types of academic schools, while the vocational high schools include agricultural, technical, commercial schools and so on. According to college status at the last follow-up (aged 18–21 years), subjects were classified as ‘college students’ or ‘non-college students.’ The environmental characteristics were the number of smoking peers at baseline (classified as ‘none’ or ‘more than one’, and ‘almost none’) and number of days not supervised by a guardian after school per week at baseline (classified as ‘1–2 days’, or ‘ > 3 days’, respectively).

Intrapersonal factors included school adjustment, alcohol drinking experience within a year, experience with health-related educational activities, and mobile phone dependency. School adjustment was measured using a 5-item survey with four-point response scales (Supplementary Table S1). We transformed the response data so that higher scores reflected more successful adjustment (at baseline). For this variable, the subjects were classified into tertile groups (low/middle/high) which ranged from less than 13, 13–14, to 15 and above. For alcohol drinking experience, the subjects were asked if they had ever drank alcohol within a year. Alcohol drinking experience at baseline was applied as a dichotomous variable (yes/no) for this study. Experience with health-related educational activities was included to examine the effects of such activities during early adolescence. Youth extracurricular activities reflect experiential learning occurring within the school environment; students typically voluntarily participate in such activities [26]. For this study, experience with health-related educational activities at baseline was dichotomized (yes/no). Mobile phone dependency was measured using a 7-item survey with four-point response scales (Supplementary Table S1). We transformed the response data so that higher scores reflected greater dependency. Mobile phone dependency questionnaire was developed and validated by Lee et al*.* (2002) [27]. To examine the third hypothesis, we used two models of mobile phone dependency. In Model 1, we added all responses at baseline, and classified subjects into tertile groups (low/medium/high) which ranged from less than 14, 14–17, to 18 and above. In Model 2, we added all responses for each wave, and used the trajectories of mobile phone dependency identified by the GBTM. Thus, we explored the association between trajectories of mobile phone dependency and smoking throughout the life-course of adolescents and young adults.

### Statistical analysis

To address missing covariate data and compare two multinomial logistic analysis models, we conducted multiple imputation analyses. Multiple imputation increases analysis efficiency and obtains unbiased estimates of the association between outcome and predictor variables [28]. The proportion of missing data at baseline ranged from 0.1% to 5.0% among variables, including family income (*n* = 90), type of high school (*n* = 17), number of days not supervised by a guardian after school (*n* = 53), experience of health-related education (*n* = 1), and mobile dependency (*n* = 83). Using the traditional listwise deletion method, about 6–10% of the 1,723 samples would have been excluded.

A frequency analysis of individual characteristics and differences in covariates by smoking trajectory group was conducted. A group-based approach was used to identify distinct developmental trajectory groups of tobacco use. For the GBTM analysis, data from wave 1 (which did not include a survey of smoking status) were excluded. PROC TRAJ, a macro in SAS software (SAS Institute, Cary, NC, USA), was used for the GBTM analysis. Because most of the subjects were nonsmokers and the distribution of the outcome data was skewed, we used ZIP for the smoking trajectory analysis [9, 29]. Furthermore, to identify life-course trajectory groups of mobile phone dependency from adolescence to young adulthood, we used censored normal distribution (CNORM), which is appropriate for continuous data. To identify the optimal number of trajectory groups and best-fitting model, we used Bayesian information criterion (BIC) values as a measure of goodness-of-fit. We selected the model with the lowest negative BIC value [9, 30].

Lastly, multinomial logistic regression analyses were conducted to identify the associations between covariates and smoking trajectory groups. We applied the longitudinal weights from the last survey wave to adjust for attrition and sample non-representativeness. Using the PROC SURVEYLOGISTIC procedure of SAS, the weighted odds ratios (OR) between covariates and smoking trajectories were calculated. In all multinomial regression analyses, multiple imputation was performed and average estimates for five imputed data sets were obtained. Using the PROC MI procedure in SAS, we created five imputed data sets. For each set, multinomial logistic regression analyses were conducted using PROC SURVEYLOGISTIC, and PROC MIANALYZE was used to combine the estimates and generate final averaged parameter estimates [28]. Model 1 examined the associations between predictors at baseline and smoking trajectory groups. Model 2 controlled for predictors at baseline, except the trajectory groups of mobile phone dependency. The trajectory of mobile phone dependency was included in Model 2. To compare the results, complete case analyses were also conducted. Further information on the missing covariates is given in the Supplementary Material (Supplementary Tables S2, S3 and S4).

## Results

Table 1 lists the characteristics of the full sample by smoking trajectory group. Among the study population, about 51% were female, and 80% went to a general high school. About 38% of the respondents were in the highest family income group, and about 39% had successful school adjustment at baseline. About 4% had alcohol drinking experience, and about 23% had more than one smoking peer at baseline. About 36% indicated that they spent more than 3 days not supervised by guardian after school, and about 17% had participated in health-related educational activities at baseline. In the final wave, about 73% of the respondents were college students. Lastly, about 37% were in the highest mobile phone dependency group at baseline. For trajectories of mobile phone dependency, about 19% of the subjects were in the highest mobile phone dependency group from adolescence to young adulthood.

**Table 1:** General characteristics of the study population by smoking trajectory group

	Total	Never smokers	Persistent light smokers	Late escalators	Early established smokers
		N (%)	N (%)	N (%)	N (%)
Total	1723 (100.0)	1290 (74.9)	79 (4.6)	151 (8.8)	203 (11.8)
Age, y (w2) (mean ± SD)	13.9 ± 0.43	13.9 ± 0.35	13.9 ± 0.43	13.9 ± 0.32	13.9 ± 0.29
Gender (w2)					
Girls	870 (50.5)	778 (60.3)	25 (31.7)	46 (30.5)	21 (10.3)
Boys	853 (49.5)	512 (39.7)	54 (68.4)	105 (69.5)	182 (89.7)
Family income (w2) ^a^					
T1	569 (33.6)	414 (32.1)	23 (28.9)	52 (34.3)	80 (39.2)
T2	507 (29.4)	380 (29.4)	25 (31.9)	41 (27.4)	61 (29.9)
T3	647 (37.6)	496 (38.4)	31 (39.2)	58 (38.3)	63 (30.9)
Type of high school (w4) ^a^					
General	1374 (79.7)	1056 (81.9)	60 (76.0)	127 (84.4)	131 (64.3)
Vocational	349 (20.3)	234 (18.1)	19 (24.1)	24 (15.6)	72 (35.7)
College status (w7)					
College students	1255 (72.8)	968 (75.0)	59 (74.7)	109 (72.2)	119 (58.6)
Non-college students	468 (27.2)	322 (25.0)	20 (25.3)	42 (27.8)	84 (41.4)
Number of days not supervised by a guardian after school (w2) ^a^					
Almost none	923 (53.6)	708 (54.9)	41 (52.2)	83 (55.0)	90 (44.2)
1–2 days	193 (11.2)	145 (11.2)	6 (7.6)	17 (11.3)	25 (12.4)
≥ 3 days	607 (35.2)	436 (33.8)	32 (40.3)	51 (33.8)	88 (43.3)
Smoking friends (w2)					
0	1333 (77.4)	1061 (82.3)	50 (63.3)	108 (71.5)	114 (56.2)
1 ≤	390 (22.6)	229 (17.8)	29 (36.7)	43 (28.5)	89 (43.8)
Drinking experience (w2)					
No	1648 (95.7)	1257 (97.4)	70 (88.6)	149 (98.7)	172 (84.7)
Yes	75 (4.4)	33 (2.6)	9 (11.4)	2 (1.3)	31 (15.3)
School adjustment (w2)					
T1	367 (21.3)	238 (18.5)	20 (25.3)	33 (21.9)	76 (37.4)
T2	682 (39.6)	528 (40.9)	26 (32.9)	56 (37.1)	72 (35.5)
T3	674 (39.1)	524 (40.6)	33 (41.8)	62 (41.1)	55 (27.1)
Experience of health-related education (w2) ^a^					
No	1427 (82.8)	1069 (82.9)	66 (83.5)	125 (82.6)	167 (82.3)
Yes	296 (17.2)	221 (17.1)	13 (16.5)	26 (17.4)	36 (17.7)
Mobile phone dependency (w2) ^a^					
T1	525 (30.5)	394 (30.6)	21 (26.3)	55 (36.4)	55 (26.9)
T2	567 (32.9)	444 (34.4)	26 (32.4)	38 (25.3)	59 (29.2)
T3	631 (36.6)	451 (35.0)	33 (41.3)	58 (38.3)	89 (43.9)
Mobile phone dependency trajectory					
Group1	384 (22.3)	305 (23.6)	10 (12.7)	40 (26.5)	29 (14.3)
Group2	1020 (59.2)	766 (59.4)	50 (63.3)	84 (55.6)	120 (59.1)
Group3	319 (18.5)	219 (17.0)	19 (24.1)	27 (17.9)	54 (26.6)

^a^ Multiple imputation analysis was applied in cases of missing values. Numbers and percentages are averages from multiple datasets

**Fig. 1:**
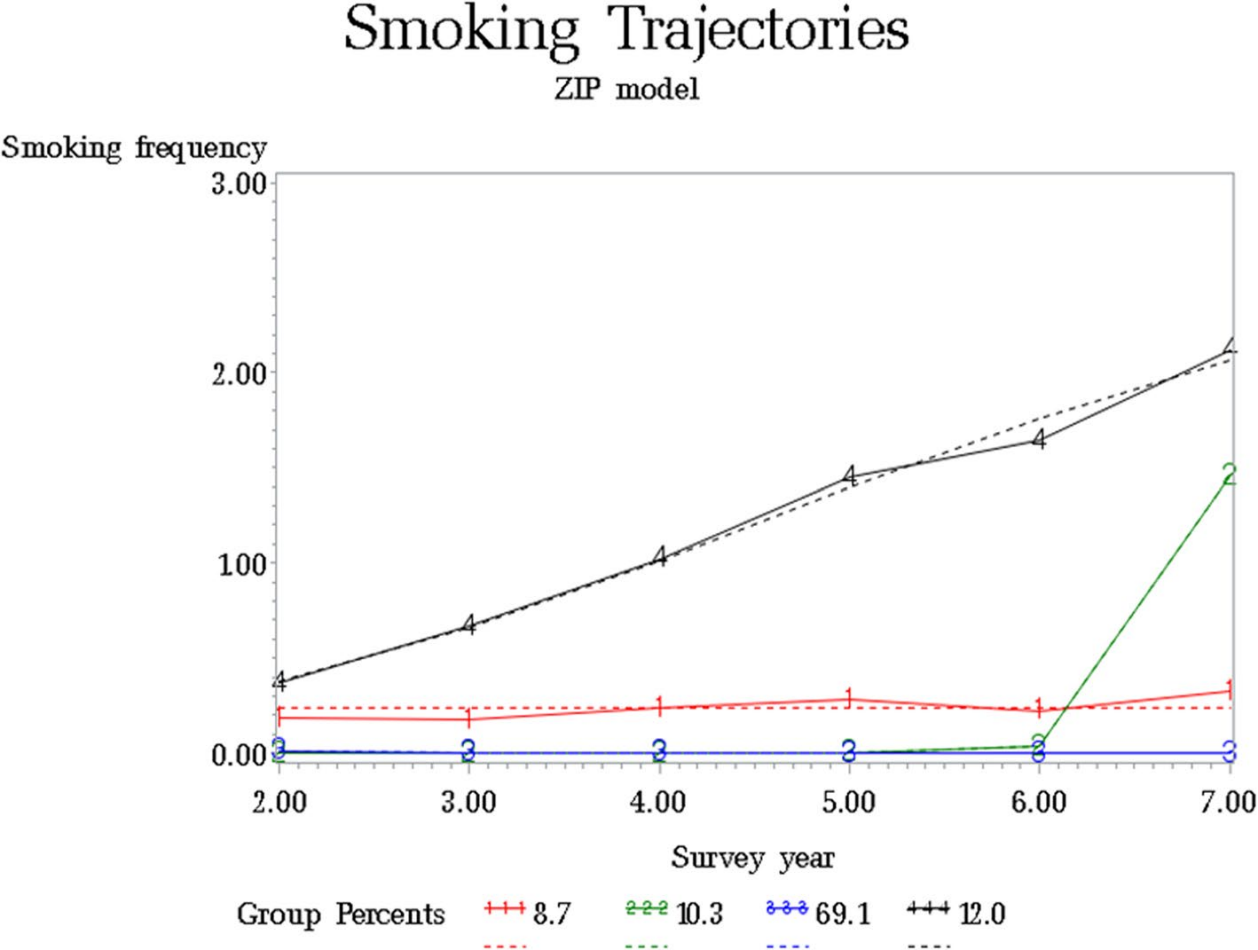
Four trajectories of smoking from early adolescence to emerging young adulthood. Note. Smoking frequency was classified as follows: 0 = none, 1 = occasional smoker in a given year, 2 = 1–9 times per day, 3 =  > 10 times per day

Figure 1 shows the smoking trajectories from adolescence to emerging young adulthood. GBTM identified four distinct smoking trajectories: never smokers (69.1%), persistent light smokers (8.7%), early established smokers (12.0%), and late escalators (10.3%), who began smoking in the 12^th^ grade (wave 6) and increased the frequency thereof in emerging adulthood (wave 7). The model fit was best for the four trajectory groups (BIC = -3,659.88). The proportion of early established smokers was second highest after never smokers. For early established smokers, smoking frequency gradually increased from middle school and peaked at 18–21 years of age. For late escalators, the smoking frequency began to increase in the final grade of high school and peaked at the age of 18–21 years.

**Fig. 2:**
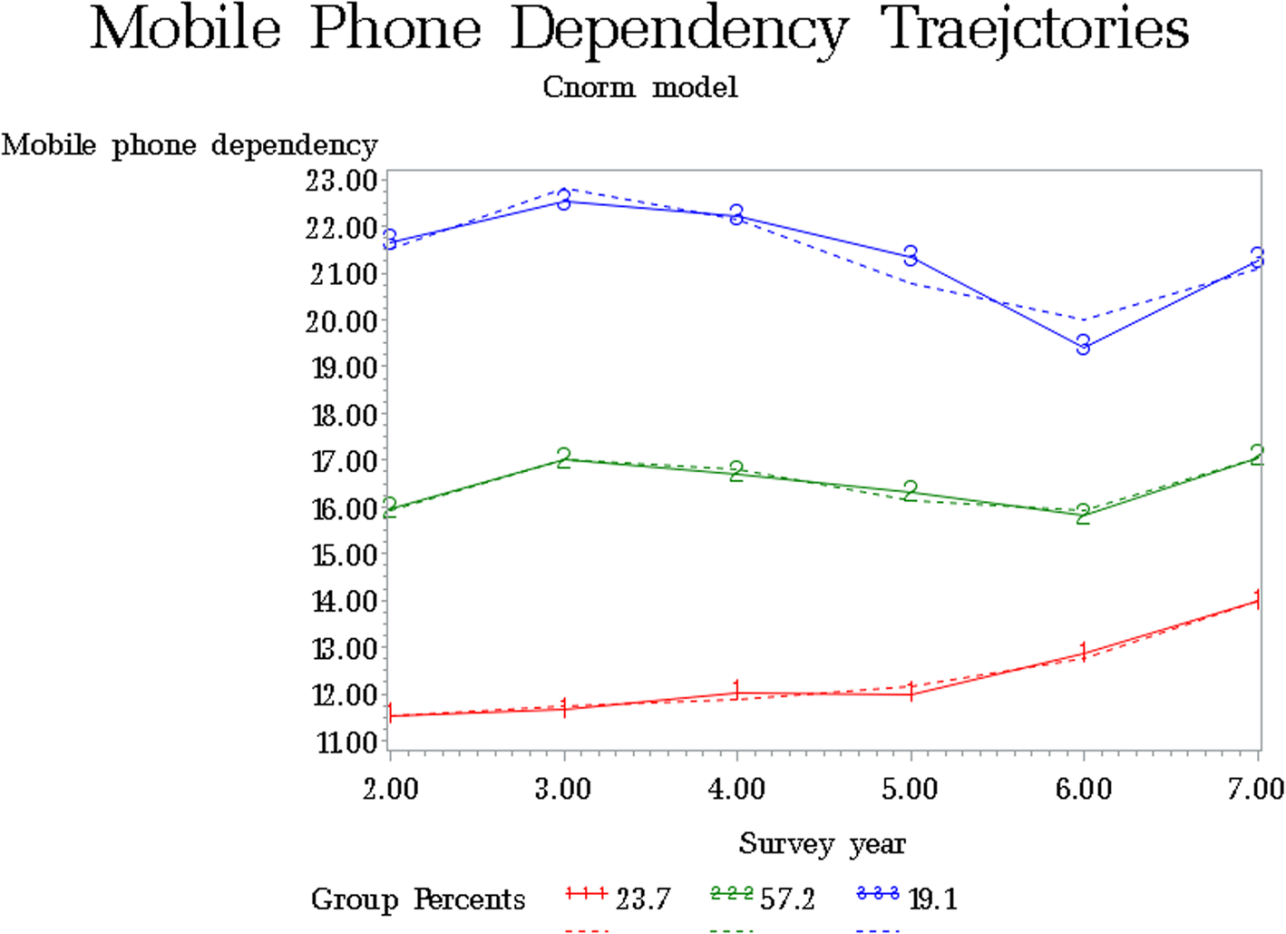
Three trajectories of mobile phone dependency from early adolescence to emerging young adulthood

Figure 2 presents the trajectories of mobile phone dependency. GBTM identified three distinct trajectories (BIC = -28,187.43): low (23.7%), middle (57.2%), and high (19.1%). For the low group, the dependency gradually increased up to adulthood. The middle and high groups showed a decreasing pattern up to the senior year of high school, and dependency increased in adulthood.

Table 2 presents the weighted ORs of covariates for smoking trajectories, calculated by multinomial logistic regression. Model 1 was conducted to examine the baseline factors associated with different trajectories of smoking, and Model 2 was used to explore the associations between the trajectories of mobile phone dependency and smoking throughout the life-course of adolescents and young adults. As shown in Model 1, compared to never smokers, boys were more likely to become any kinds of smokers than girls. Respondents who attended a vocational high school were more likely to be early established smokers (OR 2.20, 95% confidence interval [CI] 1.31–3.69). More successful school adjustment decreased the risk of being an early established smoker (OR 0.46, 95% CI 0.27–0.78). Also, subjects who spent more than 3 days per week not supervised by a guardian after school were more likely to be early established smokers (OR 1.96, 95% CI 1.23–3.13). Alcohol drinking experience increased the risk of being a persistent light smoker (OR 6.85, 95% CI 2.65–17.71) or early established smoker (OR 9.99, 95% CI 4.68–21.35). Similarly, smoking peers increased the risk of being a persistent light smoker (OR 2.33, 95% CI 1.24–4.38) or early established smoker (OR 3.01, 95% CI 1.94–4.68). Furthermore, dependency on mobile phones at baseline was associated with the risk of smoking. Subjects with greater dependency on mobile phones in early adolescence were more likely to become early established smokers (OR 3.02, 95% CI 1.75–5.21). However, family income, experience of health-related educational activities, and college status were not significantly associated with any of the smoking trajectories.

**Table 2:** Weighted odds ratios between smoking trajectories and covariates calculated by multinomial logistic regression analyses (reference trajectory group: never smokers)

	**Model 1**			**Model 2**		
	Persistent light smokers	Late escalators	Early established smokers	Persistent light smokers	Late escalators	Early established smokers
	OR (95% CI)	OR (95% CI)	OR (95% CI)	OR (95% CI)	OR (95% CI)	OR (95% CI)
Age (w2)	0.62 (0.31–1.26)	1.15 (0.63–2.12)	0.80 (0.47–1.37)	0.62 (0.31–1.25)	1.16 (0.62–2.17)	0.82 (0.49–1.38)
Gender (w2)						
Girls	1	1	1	1	1	1
Boys	3.70 (2.00–6.84)*	3.69 (2.34–5.80)*	22.32 (12.05–41.34)*	4.49 (2.31–8.73)*	4.06 (2.54–6.48)*	28.42 (14.67–55.08)*
Family income (w2)						
T1	1	1	1	1	1	1
T2	1.05 (0.51–2.17)	1.15 (0.65–2.04)	0.87 (0.52–1.44)	1.09 (0.53–2.24)	1.18 (0.65–2.12)	0.84 (0.50–1.40)
T3	0.90 (0.42–1.96)	1.04 (0.62–1.73)	0.76 (0.44–1.31)	0.90 (0.42–1.90)	1.09 (0.65–1.83)	0.72 (0.42–1.25)
Type of high school (w4)						
General	1	1	1	1	1	1
Vocational	1.54 (0.53–4.45)	0.95 (0.49–1.82)	2.20 (1.31–3.69)*	1.75 (0.75–4.08)	0.95 (0.50–1.83)	2.16 (1.28–3.66)*
College status (w7)						
College students	1	1	1	1	1	1
Non-college students	0.95 (0.48–1.89)	0.93 (0.55–1.56)	1.12 (0.69–1.81)	0.89 (0.46–1.71)	0.94 (0.55–1.58)	1.09 (0.67–1.78)
Number of days not supervised by a guardian after school (w2)						
Almost none	1	1	1	1	1	1
1–2 days	0.51 (0.20–1.27)	1.10 (0.56–2.17)	1.00 (0.48–2.09)	0.49 (0.20–1.23)	1.04 (0.53–2.05)	0.92 (0.43–1.95)
> 3 days	1.30 (0.71–2.37)	1.12 (0.70–1.80)	1.96 (1.23–3.13)*	1.24 (0.65–2.35)	1.14 (0.71–1.83)	1.78 (1.10–2.88)*
Smoking friends (w2)						
0	1	1	1	1	1	1
1 ≤	2.33 (1.24–4.38)*	1.53 (0.93–2.52)	3.01 (1.94–4.68)*	2.30 (1.21–4.38)*	1.52 (0.92–2.52)	2.94 (1.89–4.58)*
Drinking experience (w2)						
No	1	1	1	1	1	1
Yes	6.85 (2.65–17.71)*	0.43 (0.08–2.17)	9.99 (4.68–21.35)*	6.65 (2.64–16.78)*	0.44 (0.09–2.22)	10.01 (4.60–21.80)*
School adjustment (w2)						
T1	1	1	1	1	1	1
T2	0.60 (0.27–1.32)	0.63 (0.33–1.18)	0.42 (0.25–0.70)*	0.61 (0.27–1.35)	0.60 (0.32–1.14)	0.42 (0.25–0.71)*
T3	0.80 (0.37–1.72)	0.99 (0.53–1.87)	0.46 (0.27–0.78)*	0.84 (0.39–1.84)	0.99 (0.52–1.87)	0.48 (0.27–0.83)*
Experience of health-related education (w2)						
No	1	1	1	1	1	1
Yes	0.67 (0.32–1.41)	1.16 (0.66–2.04)	0.83 (0.49–1.42)	0.67 (0.32–1.42)	1.15 (0.66–2.01)	0.80 (0.47–1.36)
Mobile phone dependency						
T1	1	1	1	1	1	1
T2	1.33 (0.60–2.91)	0.58 (0.33–1.02)	1.78 (1.02–3.09)*			
T3	1.81 (0.84–3.91)	1.01 (0.61–1.65)	3.02 (1.75–5.21)*			
Mobile phone dependency trajectory						
Group1				1	1	1
Group2				2.03 (0.78–5.28)	1.13 (0.65–1.95)	3.18 (1.80–5.62)*
Group3				4.04 (1.32–12.34)*	1.61 (0.81–3.18)	8.18 (4.04–16.56)*

* *p* < 0.05

In Model 2, the effects of covariates at baseline were similar to those in Model 1. To examine the cumulative effects of mobile phone dependency, we examined the association between the trajectories of mobile phone dependency and smoking throughout the life-course of adolescents and young adults. The trajectory of mobile phone dependency was associated with the risk of being a persistent light smoker (OR 4.04, 95% CI 1.32–12.34) or early established smoker (OR 8.18, 95% CI 4.04–16.56). The effect was greatest in the most mobile phone-dependent group.

## Discussion

Based on nationally representative data, we conducted GBTM to identify developmental smoking trajectories and the associated risk factors from adolescence to emerging young adulthood. Four distinct smoking trajectories over a 6-year period were identified: never smokers, persistent light smokers, early established smokers, and late escalators.

Our findings indicate distinctive developmental smoking trajectories from adolescence to young adulthood. Smoking trajectories began to diverge during the early middle school period. The early established smokers showed an increasing smoking frequency until emerging adulthood. Late escalators began smoking in the 12^th^ grade and rapidly increased their smoking frequency in emerging adulthood. This indicates the importance of tobacco control policies and interventions for emerging young adults. Although this study included only 1 year of data for emerging young adults, late escalators generally continue to be heavy smokers until the age of 25–30 years [4, 31]. Therefore, tobacco control policies and interventions should target not only early adolescence, but also young adulthood.

Gender, type of high school, smoking peers, school adjustment, number of days not supervised by a guardian after school, alcohol drinking experience, and dependency on mobile phones in early adolescence were associated with one or more developmental smoking trajectories. Boys, students in vocational high school, and students with drinking experience and a large number of smoking peers were more likely to show early established smoking. Those well known risk factors increased the risk of smoking initiation from the early adolescence. In contrast, students with more successful school adjustment at early adolescence had a decreased risk of being early established smokers [32]. In other words, they are less likely to be regular smokers in later life. Our results indicate that predictors in early adolescence also influence smoking patterns in young adulthood.

Furthermore, more days not under the supervision of a guardian after school during early adolescence increased the risk of early established smoking. After-school supervision and the degree of self-care are associated with the risk of adolescent tobacco use [33, 34]. A randomized controlled trial showed that voluntary after-school programs may prevent alcohol use among early adolescents by promoting positive social development [35]. Our findings support the importance of after-school supervision in early adolescence, and suggest that school-based programs should aim to enhance parental monitoring; after-school programs are also needed to prevent smoking during the critical developmental period [36, 37].

To our knowledge, this is the first study of the association between mobile phone dependency and smoking trajectory. Unlike the effects of drinking alcohol and smoking peers on smoking behaviors, little is known regarding the effects of mobile phone dependency. We found positive associations between mobile phone dependency and smoking. Several cross-sectional studies support that problematic mobile phone use is associated with earlier and more extensive adolescent substance use including smoking, suggesting that mobile phone overuse is related to social relationships and psychological problems [18–20]. According to Huang et al*.* (2012), mobile phone use and social internet activity increased smoking among high school students [21]. The study suggested that mobile phone serves as a function of one’s network position, and the dependency increases the risk of smoking by enhancing the maintenance of social groups [18, 21]. Also, mobile phone dependency increases exposure to online images of substance use by peers and to various tobacco products, so that adolescents can build pro-smoking attitudes and use tobacco [18, 22]. The tobacco industry have used aggressive marketing tactics to induce adolescents and young people to experience various tobacco products [38]. A previous study found 107 pro-smoking apps for smartphones that can easily expose to adolescents and adults [22].

Importantly, to demonstrate the cumulative effect of mobile phone dependency, we examined the association between the trajectories of mobile phone dependency and smoking throughout the life-course of adolescents and young adults. The more dependent groups were, the risk of being persistent light smokers and early established smokers were increased. Mobile phone dependency during adolescence affected smoking patterns throughout adolescence and young adulthood. Therefore, mobile phone usage should be monitored in adolescents, and policies aiming to prevent exposure to smoking-related content on mobile devices should be strengthened.

This study had several limitations. First, due to the low smoking prevalence among girls, we could not examine gender differences in smoking trajectories. Further studies are needed to understand smoking trajectories and to assess risk factors for smoking by gender. Second, in contrast to other studies, we could not distinguish a “quitters” trajectory group. According to Dutra et al., quitters smoke on fewer days per month between 18 and 24 years of age [4], and also another study showed a smaller smoking amount and frequency from the age of 20 years [31]. Differences in measurements of tobacco use and analysis methods hamper comparison with prior works. The shorter survey waves for the young adulthood group may have affected our results and made it difficult to distinguish a quitters group. Third, since the first wave data in 2010 did not include a survey on smoking status, we used the second wave smoking status and set the second wave as the baseline for the most of independent variables so that it might influence the results. Fourth, the best fitting mobile phone dependency model distinguished four distinct trajectory groups (BIC = -28,181.86). However, we used three trajectory groups in the multinomial logistic regression analysis, given the relatively small sample size. The BIC for the three trajectory groups was -28,187.42. Lastly, due to the limitations of the survey, we could not determine the effects of tobacco control activities implemented by schools. Because health-related activities are not all related to tobacco use, determining the effects of extracurricular activities on smoking trajectory was problematic. However, pervious researches have shown that school-based tobacco control programs can prevent tobacco use by adolescents [39, 40]. Therefore, further longitudinal studies are needed to examine the effects of extracurricular school-based tobacco control programs on smoking patterns.

In conclusion, this population-based longitudinal study extends our knowledge of developmental tobacco use patterns from adolescence to emerging young adulthood. It demonstrated four distinctive developmental smoking trajectories occurring during critical periods. Furthermore, it highlights the long-term effects of mobile phone dependence in early adolescence on the likelihood of becoming an early established smoker. These findings support tobacco control interventions targeting the early middle school period and emerging young adults. Comprehensive tobacco control and prevention strategies that consider developmental smoking trajectories and risk factors over time are needed to prevent tobacco use among youths and young adults.

## Supplementary Information

**Additional file 1: Table S1.** KCYPS questionnaires on school adjustment and mobile phone dependency. **Table S2.** General characteristics of the complete cases (Model 1, *n* = 1,540). **Table S3.** General characteristics of the complete cases (Model 2, *n* = 1,618). **Table S4.** Weighted odds ratios between smoking trajectories and covariates using multinomial logistic regression analyses for complete case data.

## Data Availability

The public datasets analysed during the current study are available in the NYPI repository which can be accessed at http://www.nypi.re.kr/archive

## Abbreviations

GBTM: Group-based trajectory modelling
OR: Odds ratio
CI: Confidence interval
ZIP: Zero-inflated poisson
CNORM: Censored normal distribution
BIC: Bayesian information criterion
KCYPS: Korean Children & Youth Panel Survey
