# Supplementary material for: Developmental trajectories of tobacco use and risk factors from adolescence to emerging young adulthood: a population-based panel study
Source: BMC Public Health. 2022 Aug 29;22:1636. doi: 10.1186/s12889-022-14070-3 (PMC9425982; doi:10.1186/s12889-022-14070-3)
Supplement: Supplementary file 1 — Additional file 1: Table S1. KCYPS questionnaires on school adjustment and mobile phone dependency. Table S2. General characteristics of the complete cases (Model 1, n = 1,540). Table S3. General characteristics of the complete cases (Model 2, n = 1,618). Table S4. Weighted odds ratios between smoking trajectories and covariates using multinomial logistic regression analyses for complete case data. [file 12889_2022_14070_MOESM1_ESM.docx]

**Supplementary Material**

**Table S1. KCYPS questionnaires on school adjustment and mobile phone dependency.**

| Factor | Item # | Questionnaires | Responses |
| --- | --- | --- | --- |
| School adjustment | 1 | I have fun in school class | 1) Strongly agree 2) Somewhat agree 3) Somewhat disagree 4) Strongly disagree |
|  | 2 | I don't miss school homework |  |
|  | 3 | I am well aware of what I have learned in class |  |
|  | 4 | I ask other people (parents, teachers, or friends) when I don't know something |  |
|  | 5 | I do other things during class |  |
| Mobile phone dependency | 1 | The amount of time I spend using my mobile phone is increasing | 1) Strongly agree 2) Somewhat agree 3) Somewhat disagree 4) Strongly disagree |
|  | 2 | I feel anxious when I don't have my mobile phone with me |  |
|  | 3 | I feel anxious if I have not received a call or message in some time |  |
|  | 4 | I easily lose track of the time spent using my mobile phone |  |
|  | 5 | When I am alone without my mobile phone, I get easily bored and irritable |  |
|  | 6 | I feel isolated without my mobile phone |  |
|  | 7 | Without my mobile phone, I feel so uncomfortable, and I cannot live even for a day |  |

**Table S2. General characteristics of the complete cases (Model 1, n = 1,540).**

|  | Total | Never smokers | Persistent light smokers | Late escalators | Early established smokers |
| --- | --- | --- | --- | --- | --- |
|  |  | N (%) | N (%) | N (%) | N (%) |
| Total | 1540 (100.0) | 1156 (75.1) | 67 (4.4) | 138 (9.0) | 179 (11.6) |
| Age, y (w2) (mean ± SD) | 13.9±0.35 | 13.9±0.35 | 13.9±0.44 | 13.9±0.33 | 13.9±0.31 |
| Gender (w2) |  |  |  |  |  |
| Girls | 791 (51.4) | 709 (61.3) | 20 (29.9) | 43 (31.2) | 19 (10.6) |
| Boys | 749 (48.6) | 447 (38.7) | 47 (70.2) | 95 (68.8) | 160 (89.4) |
| Family income (w2) |  |  |  |  |  |
| T1 | 505 (32.8) | 365 (31.6) | 19 (28.4) | 47 (34.1) | 74 (41.3) |
| T2 | 455 (29.6) | 344 (29.8) | 22 (32.8) | 38 (27.5) | 51 (28.5) |
| T3 | 580 (37.7) | 447 (38.7) | 26 (38.8) | 53 (38.4) | 54 (30.2) |
| Type of high school (w4) |  |  |  |  |  |
| General | 1234 (80.1) | 952 (82.4) | 50 (74.6) | 119 (86.2) | 113 (63.1) |
| Vocational | 306 (19.9) | 204 (17.7) | 17 (25.4) | 19 (13.8) | 66 (36.9) |
| College status (w7) |  |  |  |  |  |
| College students | 405 (26.3) | 284 (24.6) | 15 (22.4) | 38 (27.5) | 68 (38.0) |
| Non-college students | 1135 (73.7) | 872 (75.4) | 52 (77.6) | 100 (72.5) | 111 (62.0) |
| Number of days not supervised by a guardian after school (w2) |  |  |  |  |  |
| Almost none | 821 (53.3) | 628 (54.3) | 33 (49.3) | 80 (58.0) | 80 (44.7) |
| 1-2 days | 174 (11.3) | 132 (11.4) | 6 (9.0) | 14 (10.1) | 22 (12.3) |
| ≥ 3 days | 545 (35.4) | 396 (34.3) | 28 (41.8) | 44 (31.9) | 77 (43.0) |
| Smoking friends (w2) |  |  |  |  |  |
| 0 | 1194 (77.5) | 951 (82.3) | 43 (64.2) | 95 (68.8) | 105 (58.7) |
| 1≤ | 346 (22.5) | 205 (17.7) | 24 (35.8) | 43 (31.2) | 74 (41.3) |
| Drinking experience (w2) |  |  |  |  |  |
| No | 1474 (95.7) | 1127 (97.5) | 58 (86.6) | 136 (98.6) | 153 (85.5) |
| Yes | 66 (4.3) | 29 (2.5) | 9 (13.4) | 2 (1.5) | 26 (14.5) |
| School adjustment (w2) |  |  |  |  |  |
| T1 | 312 (20.3) | 205 (17.7) | 16 (23.9) | 29 (21.0) | 62 (34.6) |
| T2 | 618 (40.1) | 475 (41.1) | 23 (34.3) | 52 (37.7) | 68 (38.0) |
| T3 | 610 (39.6) | 476 (41.2) | 28 (41.8) | 57 (41.3) | 49 (27.4) |
| Experience of health-related education (w2) |  |  |  |  |  |
| No | 1272 (82.6) | 957 (82.8) | 55 (82.1) | 113 (81.9) | 147 (82.1) |
| Yes | 268 (17.4) | 199 (17.2) | 12 (17.9) | 25 (18.1) | 32 (17.9) |
| Mobile phone dependency (w2) |  |  |  |  |  |
| T1 | 466 (30.3) | 350 (30.3) | 17 (25.4) | 50 (36.2) | 49 (27.4) |
| T2 | 500 (32.5) | 392 (33.9) | 22 (32.8) | 33 (23.9) | 53 (29.6) |
| T3 | 574 (37.3) | 414 (35.8) | 28 (41.8) | 55 (39.9) | 77 (43.0) |

For Model 1, the total number of complete cases, with listwise deletion applied for missing covariates, was 1,540. About 10% of the samples would have been excluded without multiple imputation.

**Table S3. General characteristics of the complete cases (Model 2, n = 1,618).**

|  | Total | Never smokers | Persistent light smokers | Late escalators | Early established smokers |
| --- | --- | --- | --- | --- | --- |
|  |  | N (%) | N (%) | N (%) | N (%) |
| Total | 1618 (100.0) | 1217 (75.22) | 68 (4.2) | 140 (8.7) | 193 (11.9) |
| Age, y (w2) |  |  |  |  |  |
| Gender (w2) |  |  |  |  |  |
| Girls | 818 (50.6) | 733 (60.2) | 22 (32.4) | 44 (31.4) | 19 (9.8) |
| Boys | 800 (49.4) | 484 (39.8) | 46 (67.7) | 96 (68.6) | 174 (90.2) |
| Family income (w2) |  |  |  |  |  |
| T1 | 530 (32.8) | 386 (31.7) | 19 (27.9) | 46 (32.9) | 79 (40.9) |
| T2 | 477 (29.5) | 359 (29.5) | 23 (33.8) | 40 (28.6) | 55 (28.5) |
| T3 | 611 (37.8) | 472 (38.8) | 26 (38.2) | 54 (38.6) | 59 (30.6) |
| Type of high school (w4) |  |  |  |  |  |
| General | 1296 (80.1) | 1000 (82.2) | 50 (73.5) | 120 (85.7) | 126 (65.3) |
| Vocational | 322 (19.9) | 217 (17.8) | 18 (26.5) | 20 (14.3) | 67 (34.7) |
| College status (w7) |  |  |  |  |  |
| College students | 1197 (74.0) | 923 (76.8) | 52 (76.5) | 101 (72.1) | 121 (62.7) |
| Non-college students | 421 (26.0) | 294 (24.2) | 16 (23.5) | 39 (27.9) | 72 (37.3) |
| Number of days not supervised by a guardian after school (w2) |  |  |  |  |  |
| Almost none | 868 (53.7) | 667 (54.8) | 35 (51.5) | 78 (55.7) | 88 (45.6) |
| 1-2 days | 182 (11.3) | 136 (11.2) | 6 (8.8) | 16 (11.4) | 24 (12.4) |
| ≥ 3 days | 568 (35.1) | 414 (34.0) | 27 (39.7) | 46 (32.9) | 81 (42.0) |
| Smoking friends (w2) |  |  |  |  |  |
| 0 | 1251 (77.3) | 997 (81.9) | 43 (63.2) | 97 (69.3) | 114 (59.1) |
| 1≤ | 367 (22.7) | 220 (18.1) | 25 (36.8) | 43 (30.7) | 79 (40.9) |
| Drinking experience (w2) |  |  |  |  |  |
| No | 1550 (95.8) | 1187 (97.5) | 59 (86.8) | 138 (98.6) | 166 (86.0) |
| Yes | 68 (4.2) | 30 (2.5) | 9 (13.2) | 2 (1.4) | 27 (14.0) |
| School adjustment (w2) |  |  |  |  |  |
| T1 | 327 (20.2) | 216 (17.8) | 16 (23.5) | 29 (20.7) | 66 (34.2) |
| T2 | 649 (40.1) | 503 (41.3) | 22 (32.4) | 52 (37.1) | 72 (37.3) |
| T3 | 642 (39.7) | 498 (40.9) | 30 (44.1) | 59 (42.1) | 55 (28.5) |
| Experience of health-related education (w2) |  |  |  |  |  |
| No | 1335 (82.5) | 1006 (82.7) | 57 (83.8) | 116 (82.9) | 156 (80.8) |
| Yes | 283 (17.5) | 211 (17.4) | 11 (16.2) | 24 (17.1) | 37 (19.2) |
| Mobile phone dependency trajectory |  |  |  |  |  |
| Group 1 | 314 (19.4) | 248 (20.4) | 6 (8.8) | 32 (22.9) | 28 (14.5) |
| Group 2 | 978 (60.4) | 742 (61.0) | 43 (63.2) | 79 (56.4) | 114 (59.1) |
| Group 3 | 326 (20.2) | 227 (18.7) | 19 (27.9) | 29 (20.7) | 51 (26.4) |

For Model 2, the total number of complete cases, with listwise deletion applied for missing covariates, was 1,618. About 6% of the samples would have been excluded without multiple imputation.

|  | **Model 1 (n = 1,540)** | | | **Model 2 (n = 1,618)** | | |
| --- | --- | --- | --- | --- | --- | --- |
|  | Persistent light smokers | Late escalators | Early established smokers | Persistent light smokers | Late escalators | Early established smokers |
|  | OR (95% CI) | OR (95% CI) | OR (95% CI) | OR (95% CI) | OR (95% CI) | OR (95% CI) |
| Age, y (w2) | 0.66 (0.31-1.42) | 1.12 (0.59-2.12) | 0.76 (0.43-1.34) | 0.68 (0.32-1.41) | 1.23 (0.64-2.38) | 0.75 (0.45-1.27) |
| Gender (w2) |  |  |  |  |  |  |
| Girls | 1 | 1 | 1 | 1 | 1 | 1 |
| Boys | 3.87 (1.95-7.76)* | 3.50 (2.18-5.63)* | 22.47 (11.60-43.54)* | 4.05 (1.94-8.42)* | 3.59 (2.22-5.79)* | 26.91 (13.56-53.40)* |
| Family income (w2) |  |  |  |  |  |  |
| T1 | 1 | 1 | 1 | 1 | 1 | 1 |
| T2 | 0.97 (0.48-2.00) | 1.10 (0.60-2.01) | 0.74 (0.43-1.27) | 1.05 (0.52-2.14) | 1.26 (0.70-2.28) | 0.72 (0.43-1.20) |
| T3 | 0.69 (0.33-1.44) | 1.03 (0.60-1.77) | 0.71 (0.40-1.27) | 0.69 (0.34-1.40) | 1.13 (0.67-1.92) | 0.67 (0.39-1.16) |
| Type of high school (w4) * |  |  |  |  |  |  |
| General | 1 | 1 | 1 | 1 | 1 | 1 |
| Vocational | 2.10 (1.02-4.31)* | 0.95 (0.46-1.97) | 2.84 (1.63-4.97)* | 1.88 (0.92-3.85) | 0.87 (0.44-1.73) | 2.42 (1.39-4.22)* |
| College status (w7) |  |  |  |  |  |  |
| College students | 1 | 1 | 1 | 1 | 1 | 1 |
| Non-college students | 0.63 (0.30-1.34) | 0.94 (0.54-1.64) | 0.87 (0.52-1.46) | 0.67 (0.32-1.42) | 1.06 (0.62-1.80) | 0.88 (0.53-1.46) |
| Number of days not supervised by a guardian after school (w2) |  |  |  |  |  |  |
| Almost none | 1 | 1 | 1 | 1 | 1 | 1 |
| 1-2 days | 0.68 (0.28-1.69) | 0.90 (0.43-1.87) | 0.91 (0.42-1.99) | 0.65 (0.27-1.58) | 1.11 (0.56-2.19) | 0.92 (0.44-1.91) |
| ≥ 3 days | 1.43 (0.75-2.73) | 0.98 (0.60-1.61) | 1.88 (1.17-3.04)* | 1.28 (0.67-2.44) | 1.07 (0.66-1.74) | 1.62 (1.02-2.56)* |
| Smoking friends (w2) |  |  |  |  |  |  |
| 0 | 1 | 1 | 1 | 1 | 1 | 1 |
| 1≤ | 2.08 (1.09-3.98)* | 1.68 (1.00-2.83)* | 2.77 (1.73-4.44)* | 1.96 (1.01-3.79)* | 1.63 (0.97-2.73) | 2.49 (1.59-3.91)* |
| Drinking experience (w2) |  |  |  |  |  |  |
| No | 1 | 1 | 1 | 1 | 1 | 1 |
| Yes | 9.72 (3.69-25.58)* | 0.47 (0.09-2.43) | 9.73 (4.19-22.61)* | 8.84 (3.51-22.25)* | 0.47 (0.09-2.39) | 9.51 (4.19-21.57)* |
| School adjustment (w2) |  |  |  |  |  |  |
| T1 | 1 | 1 | 1 | 1 | 1 | 1 |
| T2 | 0.83 (0.37-1.88) | 0.66 (0.34-1.30) | 0.45 (0.26-0.80)* | 0.87 (0.39-1.96) | 0.64 (0.33-1.24) | 0.48 (0.28-0.83)* |
| T3 | 1.08 (0.50-2.32) | 0.98 (0.50-1.93) | 0.47 (0.26-0.84)* | 1.26 (0.59-2.68) | 1.04 (0.53-2.06) | 0.53 (0.30-0.93)* |
| Experience of health-related education (w2) |  |  |  |  |  |  |
| No | 1 | 1 | 1 | 1 | 1 | 1 |
| Yes | 0.83 (0.37-1.85) | 1.18 (0.66-2.11) | 0.90 (0.51-1.58) | 0.76 (0.33-1.74) | 1.12 (0.63-2.01) | 0.92 (0.54-1.55) |
| Mobile phone dependency |  |  |  |  |  |  |
| T1 | 1 | 1 | 1 |  |  |  |
| T2 | 1.40 (0.62-3.20) | 0.47 (0.26-0.85)* | 1.77 (1.00-3.11)* |  |  |  |
| T3 | 1.76 (0.81-3.85) | 0.92 (0.55-1.54) | 2.64 (1.50-4.62)* |  |  |  |
| Mobile phone dependency  trajectory |  |  |  |  |  |  |
| Group1 |  |  |  | 1 | 1 | 1 |
| Group2 |  |  |  | 2.50 (0.84-7.44) | 1.06 (0.58-1.94) | 2.19 (1.21-3.95)* |
| Group3 |  |  |  | 5.09 (1.54-16.85)* | 1.57 (0.78-3.19) | 4.95 (2.44-10.07)* |

**Table S4.** Weighted odds ratios between smoking trajectories and covariates using multinomial logistic regression analyses for complete case data.
